# Research status and prospects of pituitary adenomas in conjunction with neurological and psychiatric disorders and the tumor microenvironment

**DOI:** 10.3389/fnins.2024.1294417

**Published:** 2024-04-22

**Authors:** Siyuan Cui, Sainan Chen, Xuechao Wu, Qing Wang

**Affiliations:** ^1^Department of Endocrinology, Jiangnan University Medical Center, Wuxi No.2 People’s Hospital, Wuxi, China; ^2^Department of Endocrinology, Wuxi No.2 People’s Hospital, Affiliated Wuxi Clinical College of Nantong University, Wuxi, China; ^3^School of Clinical Medicine, Nanjing Medical University, Nanjing, Jiangsu, China; ^4^Department of Neurosurgery, Jiangnan University Medical Center, Wuxi No.2 People’s Hospital, Wuxi, Jiangsu, China; ^5^Wuxi Neurosurgical Institute, Wuxi, Jiangsu, China; ^6^Neuroscience Center, Wuxi School of Medicine, Jiangnan University, Wuxi, Jiangsu, China

**Keywords:** pituitary neuroendocrine tumors, neuropsychiatric disorders, tumor microenvironment, cognitive impairment, depression, anxiety, immune cells, cytokines

## Abstract

Patients with pituitary neuroendocrine tumors (PitNETs) often experience neuropsychiatric disorders due to factors such as hormonal imbalances, and inadequate management of medications, surgeries, and radiation therapies. Commonly observed disorders include depression, anxiety, and cognitive dysfunction, which significantly impact patients’ quality of life and prognosis. PitNETs have a significant presence of immune cells within the tumor microenvironment (TME), predominantly macrophages and T lymphocytes. These immune cells secrete a variety of cytokines, growth factors, and chemokines, which regulate the biological behaviors of PitNETs, including tumor initiation, proliferation, migration, invasion, and angiogenesis. In addition, this review provides a pioneering summary of the close relationships between the aberrant secretion of proinflammatory cytokines within the TME of PitNETs and the occurrence of neuropsychiatric disorders, along with their potential underlying mechanisms. The cytokines produced as a result of TME dysregulation may affect various aspects of the central nervous system, including neurotransmitter metabolism, neuroendocrine function, and neurovascular plasticity, thereby leading to a higher susceptibility to neurobehavioral disorders in PitNET patients.

## 1 Introduction

Pituitary adenomas (PAs), which occur in the pituitary gland, are common tumors of the nervous and endocrine systems, accounting for approximately 10%–15% of all intracranial tumors. In the 5th edition of Mete and Wenig (2022), pituitary adenomas were renamed PitNETs. Recent studies have interestingly found that PitNET patients are prone to severe neurological and psychiatric disorders. These disorders significantly increase the diagnostic and treatment challenges for PitNET patients and greatly affect their quality of life. Drastic changes in the tumor microenvironment (TME) are one of the important mechanisms underlying the occurrence and development of PitNETs. The occurrence of neurological and psychiatric disorders in PitNET patients is likely associated with the disruption of the TME. To better understand the occurrence of neurological and psychiatric disorders in patients with PitNETs and the potential disruption of the TME, a retrospective analysis and comprehensive summary of scientific research on PitNETs was conducted, with a specific focus on examining the relationship between these disorders and the TME within PitNETs.

## 2 PitNETs

### 2.1 Diagnosis and detection methods for PitNETs

Pituitary neuroendocrine tumors (PitNETs), mostly benign tumors, can cause corresponding clinical symptoms due to compression by the tumor mass. These symptoms may include headaches, visual impairment, increased intracranial pressure, hypopituitarism, or a series of adverse effects caused by excessive or insufficient hormone secretion (Di Ieva et al., 2014; Trouillas et al., 2020). Approximately 10% of PitNETs exhibit invasive behavior, such as invading the cavernous sinus and/or the sphenoid sinus. The vast majority of PitNETs are sporadic, with only 5% being familial cases (Marques and Korbonits, 2017).

The diagnosis of PitNETs primarily relies on the patient’s medical history, symptoms, physical examination, endocrine test, and radiological imaging findings. Among them, magnetic resonance imaging (MRI) and visual field examination are used to accurately locate tumors and assess local tumor compression (Melmed, 2020). Endocrine tests are conducted to evaluate hormone secretion and differentiate functional and nonfunctional adenomas, as well as to assess pituitary reserve function (Freda et al., 2011). Immunocytochemical evaluation of differentiated, cell-specific pituitary transcription factors and hormones, along with precise biochemical, imaging, and clinical phenotyping, elucidate tumor characteristics and endocrine syndromes. This comprehensive approach enables tailored treatments for individuals with distinct endocrine syndromes (Melmed, 2020).

### 2.2 The latest classification of PitNETs

Currently, based on the measurement of tumor diameter using MRI, PitNETs are classified into microadenomas (<10 mm), macroadenomas (10–40 mm), and giant adenomas (>40 mm). Additionally, based on hormone secretion, PitNETs are further classified into functional PitNETs and nonfunctioning pituitary neuroendocrine tumors (NF-PitNETs) (Melmed, 2020; Trouillas et al., 2020). Functional PitNETs exhibit excessive hormone secretion, leading to specific clinical manifestations. For example, elevated levels of growth hormone (GH) can cause acromegaly or gigantism, increased levels of prolactin (PRL) can result in amenorrhea-galactorrhea syndrome in females or sexual dysfunction in males, and hypercortisolism can cause Cushing’s disease (CD) (Trouillas et al., 2020). NF-PitNETs are more commonly observed in middle-aged men and postmenopausal women. The clinical features include headache, visual field defects, and hypopituitarism caused by the compression of the optic chiasm and pituitary tissue by the tumor. Except for large-sized NF-PitNETs that can cause hyperprolactinemia due to the compression of the pituitary stalk, NF-PitNETs do not exhibit clinical manifestations caused by hormone hypersecretion (Manojlovic-Gacic et al., 2018; Melmed, 2020).

In the latest 2022 WHO classification, PitNETs are categorized into different lineages based on immunohistochemical detection of pituitary transcription factors (TFs) in adenohypophyseal cells. According to the different cell lineage, PitNETs are classified as follows (Table 1): 1. Pituitary-specific transcription factor-1 (PIT-1) lineage: GH adenomas, PRL adenomas, lactotroph growth hormone cell adenomas (cells that can secrete both hormones), mixed GH-PRL cell adenomas (cell mixtures that secrete GH and PRL, respectively), thyroid-stimulating hormone (TSH) cell adenomas, and acidophilic stem cell adenomas (cells with focal acidophilic changes in the cytoplasm); 2. T-box family member TBX19 (also known as TPIT) lineage: ACTH adenomas; 3. Steroidogenic factor-1 (SF-1) lineage: gonadotropin (Gn) cell adenomas (Asa et al., 2022); 4. Multiple hormone adenomas without specific immunohistochemical features of specialized adenohypophyseal cell differentiation and null cell (NC) adenomas (Trouillas et al., 2020). Among them, GH adenomas, PRL adenomas, and ACTH adenomas have subtypes characterized by dense granules and sparse granules, reflecting the number of cytoplasmic secretory granules. TSH adenomas and Gn cell adenomas do not have such subtypes.

**TABLE 1:** The 2022 World Health Organization classification of PitNETs.

PitNET type			Hormones	LMWK
PIT1-lineage PitNETs	Somatotroph tumors	Densely granulated somatotroph tumor	GH, α-subunit	Perinuclear
		Sparsely granulated somatotroph tumor	GH	Fibrous bodies (>70%)
PIT1-lineage PitNETs	Lactotroph tumors	Densely granulated lactotroph tumor	PRL (diffuse cytoplasmic)	Weak or negative
		Sparsely granulated lactotroph tumor	PRL (paranuclear dot-like)	Weak or negative
	Mammosomatotroph tumor		GH (predominant), PRL, α-subunit	Perinuclear
	Mixed somatotroph and lactotroph tumor		Somatotroph tumor component: GH ± α-subunit; lactotroph tumor component: PRL (diffuse or paranuclear)	Tumor subtype characteristics
	Thyrotroph tumor		α-Subunit, βTSH	Weak or negative
	Acidophil stem cell tumor		Monomorphic tumor cells with PRL (predominant) and GH (focal/variable)	Scattered fibrous bodies
	Mature plurihormonal PIT1-lineage tumor		Monomorphic tumor cells with predominant GH expression and variable PRL, βTSH, and α-subunit	Perinuclear
	Immature PIT1-lineage tumor		Focal/variable staining for no hormones, or one or more of GH, PRL, βTSH, and/or α-subunit	Focal/variable
TPIT-lineage PitNETs	Corticotroph tumors	Densely granulated corticotroph tumor	ACTH and other POMC derivatives	Strong, always diffuse
		Sparsely granulated corticotroph tumor	ACTH and other POMC derivatives	Variable (often diffuse)
TPIT-lineage PitNETs	Corticotroph tumors	Crooke cell tumor	ACTH and other POMC derivatives	Perinuclear ring-like cytoplasmic
SF1-lineage PitNETs	Gonadotroph tumor		α-Subunit, βFSH	Variable or negative
PitNETs with no distinct cell lineage	Null cell tumor		None	Variable
	Plurihormonal tumor		Multiple combinations in a monomorphous tumor population	Variable

## 3 PitNETs combined with neuropsychiatric disorders

### 3.1 Overview of neuropsychiatric disorders

Neuropsychiatric disorders encompass a wide spectrum of conditions at the intersection of neurology and psychiatry. These include depressive disorders, anxiety, schizophrenia, bipolar disorder, attention deficit hyperactivity disorder (ADHD), autism spectrum disorders, headaches, and epilepsy (Saleki et al., 2024). In addition to genetic factors and environmental stress, various diseases can contribute to the development of neuropsychiatric disorders, although the severity and clinical presentations may vary. In practical terms, chronic liver disease and acute liver failure can lead to a diverse range of neuropsychiatric abnormalities, ranging from subtle cognitive impairment to severe disorientation, confusion, and coma (Rose et al., 2020). Additionally, conditions like hypertension, autoimmune diseases, renal dysfunction/failure, and preeclampsia/eclampsia may precipitate acute or subacute neurological symptoms, including headache, seizure, confusion, vomiting, and visual disturbances (Li et al., 2023). Severe neuropsychiatric disorders have a profound impact on the quality of life of patients, significantly complicating the process of diagnosis and treatment (Cosci et al., 2015; Santos et al., 2017; Bengtsson et al., 2021; Coronel et al., 2022).

### 3.2 The occurrence and causes of neuropsychiatric disorders in patients with PitNETs

A prior study indicated that major depression affects 54%–65% of patients with active Cushing’s syndrome (CS), arising from both CD and other causes of hypercortisolism. Notably, no significant differences in depression rates have been observed between pituitary-dependent and pituitary-independent forms of CS, implying that hypercortisolism itself may be the underlying cause (Bengtsson et al., 2021). Moreover, in addition to depression, these patients frequently exhibit depression-related psychopathological symptoms, including anxiety, sleep disturbances, fatigue, cognitive impairment (primarily attention and memory deficits), and, less commonly, manic behavior (Sonino and Fava, 2001; Pereira et al., 2010; Pivonello et al., 2015; Santos et al., 2017). Furthermore, Burback (2015) documented various behavioral changes observed in four PRL adenoma patients. These changes encompassed symptoms such as low mood, irritability, and verbal aggression, as well as more severe manifestations including psychosis, mania, and paranoid delusions. Moreover, a cross-sectional study involving 153 GH adenoma patients revealed that approximately 50% of the patients experienced psychiatric disorders, with anxiety and insomnia being the most prevalent conditions (Szcześniak et al., 2017).

In fact, the prevalence of neurocognitive dysfunction ranges from 15% to 83% in ACTH adenoma patients and from 2% to 33% in GH adenoma patients (Sievers et al., 2012), with primary memory and attention being affected (Tiemensma et al., 2011). Memory changes occur in 22% of NF-PitNET patients. The prevalence of psychiatric disorders reaches 77% in ACTH adenoma patients and 63% in GH adenoma patients, mainly involving depression, followed by psychosis and anxiety (Tooze et al., 2012). The prevalence of psychopathological symptoms is as high as 83% in ACTH adenoma patients and 35% in GH adenoma patients (Tooze et al., 2012). These results indicate that PitNET patients are indeed prone to developing neuropsychiatric disorders, and the condition can fluctuate and progress rapidly. The main manifestations include anxiety, depression, and a decline in memory and attention, with the potential for developing more severe psychiatric disorders like mania, paranoid delusions, and personality changes.

Pituitary neuroendocrine tumor patients experience various underlying factors contributing to neuropsychiatric disorders, including impaired body image, forced alteration of brain structures (particularly with the expansion of invasive tumors into the frontal and temporal lobes), hormonal imbalances, reduced sexual function, and the impacts of surgical procedures, medications, and radiation therapy (Tiemensma et al., 2011).

### 3.3 The effect of tumor size on neuropsychiatric disorders in patients with PitNETs

In their report, Shi et al. (2022) described a compelling case of a patient diagnosed with GH adenoma, who presented with acute psychiatric symptoms, including hallucinations, paranoia, severe impulsivity, and aggressive behavior (Tiemensma et al., 2011). Enhanced MRI revealed a large adenoma, which compressed the optic chiasm, invaded the skull base bones and encased the vessels in the sellar region (Tiemensma et al., 2011). Following the exclusion of other organic diseases that could cause neuropsychiatric symptoms, it was determined that the patient’s psychiatric symptoms could be attributed to the GH adenoma. However, in this patient, the GH/insulin-like growth factor-1 (IGF-1) levels had remained stable for the past 3 years, and there were no changes in the size of the primary adenoma observed on brain imaging (Tiemensma et al., 2011). However, a systematic study of 76 PitNET patients found no correlation between cognitive function changes and tumor size (Siegel et al., 2016). Similarly, in a study involving 200 PitNET patients, Korali et al. (2003) also found that there was no association between tumor size and the presence of psychiatric disorders. These findings suggest that tumor size may not be a significant factor influencing the development of psychiatric conditions in PitNET patients.

However, large PitNETs may compress the lateral wall of the cavernous sinus, causing cavernous sinus syndrome. The cavernous sinus syndrome is a set of clinical signs characterized by the appearance of multiple cranial neuropathies, which include involvement of the ocular motor nerves due to a lesion of the cranial pairs III, IV, and VI, and sensory impairment in the first and second branches of the trigeminal nerve. Also, Horner’s syndrome can be present, due to the lesion of the sympathetic nervous system. According to the literature, approximately 2.4%–8.3% of pituitary adenomas can cause cavernous sinus nerve palsy, which significantly reduce the quality of life of patients (Fu et al., 2017). In a retrospective analysis of the characteristics of 70 PitNET patients with cavernous sinus syndrome who received surgical treatment, 55.7% recovered within 2 weeks of surgery, 24.3% recovered from 2 weeks to 1 year after surgery, and 20% had not returned to normal after more than 1 year after surgery (Fu et al., 2017). Cavernous sinus syndrome that persists for a long time may cause neuropsychiatric disorders in patients with pituitary adenomas but limited research is available. The evaluation of more PitNET cases in the future is necessary, especially to strengthen the comprehensive assessment of neuropsychiatric disorders in patients with invasive PitNETs.

### 3.4 The impact of drug treatment on neuropsychiatric disorders in patients with PitNETs

Dopamine agonists (DAs) are the most effective first-line drugs for treating PRL adenomas due to their functions of inhibiting PRL secretion and reducing tumor size (Shi et al., 2022), and DAs are also recommended for acromegaly with mild symptoms (Melmed and Longo, 2020). Cabergoline and bromocriptine are currently the most widely used DAs (Wang et al., 2017). The use of DAs in the treatment of PitNETs is associated with transient depression, increased libido, impulse control disorders (ICDs), or psychiatric symptoms. The impact on patient’s mental status is primarily caused by two complex mechanisms. These include the cross-stimulation of D3 receptors expressed in the mesolimbic dopamine pathway and the sudden replacement and restoration of sex hormones (especially testosterone) to normal levels in the central hypogonadism state after central nervous system dopamine depletion due to hyperprolactinemia (Bancos et al., 2014; Wang et al., 2017). Cabergoline and bromocriptine inhibit prolactin (PRL) secretion by binding to dopamine D2 receptors, while also exhibiting a certain affinity for dopamine D3 receptors. The D3 receptor is expressed in brain regions controlling reward, emotions, and motivation and plays crucial roles in regulating motor functions, addiction, cognition, schizophrenia, and other physiological processes (Sokoloff and Le Foll, 2017).

In a case-control study conducted by Hinojosa-Amaya et al. (2020) it was found that patients with PitNETs who received treatment with DAs had a higher prevalence of moderate to severe depression than PitNET patients who did not receive DA treatment (Melmed et al., 2011). Furthermore, severe depression was observed only in patients receiving DA treatment (Melmed et al., 2011). In a prospective assessment study evaluating screening for conditions such as obsessive-compulsive disorder, interpersonal sensitivity, and paranoia, it was found that the incidence rate was higher in PRL adenoma patients who received DA treatment than in those who did not receive DA treatment (Ioachimescu et al., 2019) ICDs represent a category of destructive disorders characterized by impaired impulse control and behavioral abnormalities, and these disorders can manifest in various ways, including pathological gambling, increased libido, binge eating, compulsive shopping, compulsive drug use, and compulsive hobbies (Wang et al., 2017). PRL adenoma patients receiving DA treatment have higher ICD scores than patients with hyperprolactinemia who are not treated with DAs as well as healthy control individuals. Bancos et al. (2014) found that PRL adenoma patients using DAs had a ninefold increased risk of developing ICDs than NF-PitNET patients who did not receive DA treatment. Additionally, multiple ICDs manifestations can occur simultaneously in the same patient, with pathological hypersexuality being the most prevalent. The study also revealed that there was no correlation between the presence of an ICD and the type of DA, treatment duration, or dosage. Reports by Martinkova et al. (2011) and De Sousa et al. (2017) demonstrated that symptoms of increased libido in PRL adenoma patients treated with DAs can be alleviated or resolved when the DA dosage is reduced or discontinued, which confirmed the reversibility of DA-induced ICDs. Furthermore, in PRL adenoma patients receiving DA treatment, there were more cases of increased libido in males than in females, but there was no sex difference for other ICD types (Bancos et al., 2014; Ioachimescu et al., 2019). Many studies have confirmed that DAs can cause neurological and psychiatric disorders in PRL patients. Although symptoms may improve after discontinuing the use of DAs and antipsychotic medications, it is still important for clinical doctors to pay attention to promptly and effectively assess and intervene in the neurological and psychiatric conditions of patients who are using DAs.

### 3.5 The impact of hormone levels on the occurrence of neurological and psychiatric disorders in PitNET patients

In a study conducted by Wang et al. (2017), it was found that preoperative cognitive function scores in the majority of functional PitNET patients were significantly lower than those in NF-PitNET patients and healthy control individuals. This suggests that endocrine disruption may be an important factor causing cognitive function changes. After surgery, hormone levels returned to normal in most functional PitNET patients, leading to a significant improvement in cognitive function scores (Wang et al., 2017). The improvement in cognitive function is speculated to be attributed to the reduction in hormone secretion from PitNET cells after tumor removal or the surgical relief of tumor compression on the pituitary, thereby enhancing the secretory function of normal pituitary tissue.

In a prospective research comparing GH adenoma patients and NF-PitNET patients, it was found that the preoperative and postoperative changes in GH levels were significantly correlated with changes in depressive symptoms in GH adenoma patients (Algahtany et al., 2021). However, no decrease in depression scores was observed in NF-PitNET patients after surgery. The results suggest that elevated GH levels may contribute to the occurrence of depression symptoms (Algahtany et al., 2021). There is evidence suggesting that the severity of psychiatric disorders in GH adenoma patients increases with increased hormone secretion, which is associated with the irreversible effects of the high levels of GH/IGF-1 on the central nervous system (Shi et al., 2022). A study involving 86 PRL adenoma patients confirmed that patients with high PRL levels have lower dopaminergic activity, which is associated with decreased novelty-seeking motivation and increased scores in avoidance of harm (Athanasoulia et al., 2012). Pereira et al. (2012) reported that GH adenoma and ACTH adenoma patients who have been successfully treated and experienced long-term remission have a higher prevalence of psychiatric disorders and more maladaptive personality traits. This suggests that the impact of previous PitNET-induced hormone hypersecretion on the central nervous system may be persistent and, to some extent, even irreversible. Testosterone has been found to have antidepressant and neuroprotective effects in the hippocampus, limbic system, and other brain regions involved in regulating emotions (McHenry et al., 2014; Williams et al., 2022). A large body of research indicates a significant correlation between low testosterone levels, clinical hypogonadism, pharmacological testosterone deficiency induced by androgen deprivation therapy, and treatment with androgen receptor antagonists in male depression (McIntyre et al., 2006; Almeida et al., 2008; Joshi et al., 2010). This suggests that the dysregulation of the hypothalamic-pituitary-gonadal axis plays an important role in the onset of severe depression. Furthermore, a recent longitudinal study of young healthy males (average age 34 years) found that an increase in gonadotropin secretion led to a decrease in the testosterone/luteinizing hormone ratio, indicating a decline in Leydig cell function for testosterone synthesis in the testes (Fantus et al., 2021). However, there is still limited research available on the association between testosterone levels and depression specifically in individuals with Gn (gonadotropin) adenoma. Further studies are needed to explore this potential connection.

### 3.6 The impact of surgical approaches on the presence of neurological and psychiatric disorders in PitNET patients

In an early study, PitNET patients who underwent surgical treatment (i.e., transfrontal or transsphenoidal surgery along with medication) were compared with healthy control subjects and patients who received medication alone. The results showed that both the transfrontal and transsphenoidal surgery groups exhibited more pronounced impairments in memory and executive function than the healthy control group. Patients who underwent transsphenoidal surgery reported higher levels of emotional disorders, emotional distress, poor social adaptation, and impaired daily functioning. Although patients who underwent transfrontal surgery did not differ from those who underwent transsphenoidal surgery in self-assessment, objective evaluations showed significantly worse neurological and psychiatric outcomes for these patients than for those in the transsphenoidal surgery group (Peace et al., 1997). The observed bias in the results is attributed to the impaired insight of patients who underwent transfrontal surgery, which leads to inaccurate self-assessment. This suggests that transfrontal surgeries may aggravate neurological and psychiatric disorders in PitNET patients.

However, a recent study by Wang et al. (2017) found that cognitive functions including orientation, language comprehension, memory, practical skills, abstract thinking, and perception significantly improved compared to preoperative levels in PitNET patients who underwent surgery using either the single-nostril transsphenoidal microscopic approach or the neuroendoscopic approach. However, no differences were found between the different surgical approaches. This suggests that minimally invasive surgical techniques have minimal impact on brain structural tissues in patients, and both approaches can improve cognitive function by removing the tumor and resolving the hormonal effects of tumor oversecretion.

The above research findings indicate that traditional frontal craniotomy can impair the emotions, affect, and cognitive function of PitNET patients. However, with advancements in surgical techniques, current endoscopic and microscopic transnasal transsphenoidal approaches have actually been beneficial for the recovery of cognitive impairments in PitNET patients.

### 3.7 The impact of radiation therapy on the occurrence of neurological and psychiatric disorders in patients with PitNETs

The hippocampus is a vital region in the brain that plays a crucial role in processing sensory stimuli and in the formation, consolidation, and retrieval of memories (Opitz, 2014). The temporal lobe/hippocampus and the prefrontal cortex (PFC) are radiation-sensitive areas in the brain that are associated with cognitive processes (Brummelman et al., 2012). The PFC is particularly important for executive functions, such as planning, cognitive flexibility, and inhibitory control (Miller and Cohen, 2001).

A cross-sectional comparative study of 75 NF-PitNET patients showed that current radiation therapy did not result in significant differences in cognitive performance in terms of the hippocampus and prefrontal cortex between the radiation group and the non-radiation group (Brummelman et al., 2012). Guinan et al. (1998) also reported that there were no significant differences in memory impairment between those who underwent surgery alone and those who underwent surgery combined with radiation therapy in PitNET patients, although both groups experienced anterograde amnesia. In contrast, Noad et al. (2004) found that patients who received postoperative radiation therapy had poorer executive functioning than those who underwent surgery alone. Grattan-Smith et al. (1992) also reported that both groups of PitNET patients, one receiving radiation therapy and the other not receiving radiation therapy, exhibited worse neurocognitive performance than the healthy control group. However, there were no differences in terms of executive functioning, nonverbal memory, or visual memory impairments between the two groups. The discrepancies among these studies suggest that there may be a threshold for radiation therapy-induced brain damage and cognitive impairment, where cognitive function is affected only when the threshold is exceeded. Therefore, in clinical practice, it is important for physicians to accurately delineate the target area and surrounding normal brain structures, and operate within a safe range of radiation dose, aiming to minimize radiation-induced damage while ensuring optimal tumor control.

## 4 The TME of PitNETs

### 4.1 TME

Tumor cells in PitNETs are surrounded by immune cells and non-immune cells such as stromal cells or endothelial cells. These cells interact with cytokines, chemokines, and growth factors present in the tumor’s extracellular matrix (ECM), collectively forming the TME. Different cellular components within the TME, along with the small protein molecules they secrete, play a role in regulating the tumor development, proliferation, and invasive capabilities of PitNETs (Balkwill, 2012; Balkwill et al., 2012; Drake and Macleod, 2014).

### 4.2 The TME of PitNETs

The immune infiltration in PitNETs differs from that in normal pituitary tissue. PitNETs exhibit a higher infiltration of immune cells within the TME, and the quantity and types of infiltrating immune cells vary among different types of PitNETs (Table 2). The main immune cell types found in PitNETs are macrophages and T lymphocytes, including both CD4^+^ and CD8^+^ T cells (Lu et al., 2015; Marques et al., 2019a; Iacovazzo et al., 2020; Principe et al., 2020; Wang et al., 2020; Yeung et al., 2020; Mei et al., 2021; Nie et al., 2021). Immune cells with anti-tumor effects typically include M1 macrophages, cytotoxic CD8^+^ T lymphocytes, natural killer (NK) cells, and neutrophils, while M2 macrophages, Forkhead box protein P3 (FOXP3)^+^ T cells, and CD4^+^ helper T cells are often associated with promoting tumor growth. The interactions between these immune cells vary significantly at different stages of tumor development (Mantovani et al., 2008; Balkwill and Mantovani, 2012; Marques et al., 2020b). Some studies suggest macrophages are the predominant immune cell type in PitNETs (Lu et al., 2015; Marques et al., 2019a; Principe et al., 2020; Zhang et al., 2021), while others have found that CD8^+^ T cells and CD4^+^ T cells are the most abundant immune cells in PitNETs (Wang et al., 2020; Yeung et al., 2020; Zhou et al., 2020). The inconsistent research results can be attributed to variations in the studied population, sample size, and different tumor types, indicating significant differences in immune cell infiltration among different types of PitNETs. In addition to macrophages and T lymphocytes, there is a small population of other immune cell subsets infiltrating PitNETs, including B lymphocytes, neutrophils, NK cells, and mast cells (Marques et al., 2019a; Principe et al., 2020; Taniguchi-Ponciano et al., 2020; Zhou et al., 2020; Zhang et al., 2021).

**TABLE 2:** Immune cells in the TME of PitNETs.

Main findings	References
**Immune cells: macrophages**	
• All the adenomas showed varying degrees of CD68^+^ macrophage infiltration. • The numbers of CD68^+^ macrophages were positively correlated with the tumor sizes and Knosp classification grades for tumor invasiveness. • The infiltration of CD68^+^ macrophages was significantly greater in SG-GH than in DG-GH and ACTH adenomas.	Lu et al., 2015
• The number of CD68^+^ macrophages did not differ based on tumor size, cavernous sinus invasion, or treatment responsiveness.	Iacovazzo et al., 2020
• PitNETs had more macrophages than NF-PitNETs, with a threefold increased CD163: HLA-DR macrophage ratio, which was positively correlated with microvessel density and area.	Marques et al., 2019a
• Pituitary tumors were infiltrated by macrophages and T cells.	Mei et al., 2021
• Gonadotroph PitNETs presented an increased CD68^+^ macrophage signature compared to somatotroph, lactotroph, and corticotroph PitNETs. • There was a significant correlation between the percentage of CD68^+^ and CD163^+^ infiltrating macrophages and the invasive character of gonadotroph tumors. • Transcriptomic and histological characterizations confirmed that gonadotroph infiltrating macrophages expressed CD163, mannose receptor (MRC-1), arginase 1 (ARG1), and colony-stimulating factor 1 receptor (CSF1R) M2 macrophage markers.	Principe et al., 2020
• The majority of infiltrating immune cells within pituitary adenomas were composed of M2 macrophages, followed by resting CD4^+^ memory T cells and mast cells. • NF-PitNETs had higher M2 macrophage fractions than other subtypes.	Yeung et al., 2020
• Of the 64 PitNET tissues, invasive PitNETs were related to high infiltration of M2-like tumor-associated macrophages (TAMs). • Lactate secreted from PitNET cells facilitated M2 polarization via the mTORC2 and ERK signaling pathways, while activated TAMs secreted CCL17 to promote PitNET invasion via the CCL17/CCR4/mTORC1 axis.	Zhang et al., 2021
• Both M1 and M2 macrophages were present in the anterior pituitary gland of rats. Moreover, the number of M2 macrophages was greatly increased in rats with DES-induced prolactinoma.	Fujiwara et al., 2017
**Immune cells: T-lymphocytes**	
• The number of CD8^+^ lymphocytes was significantly lower in tumors that invaded the cavernous sinus than in those without cavernous sinus invasion.	Iacovazzo et al., 2020
• The infiltration of CD4^+^ and CD8^+^ T cells was relatively scant in these adenomas, but GH adenomas exhibited significantly more CD4^+^ and CD8^+^ T cells than non-GH adenomas. Both DG-GH and SG-GH adenomas had significantly more CD4^+^ cells than ACTH adenomas and significantly more CD8^+^ cells than NC adenomas.	Lu et al., 2015
• PitNETs contained more CD4^+^ T-lymphocytes, but fewer neutrophils, with a twofold decreased CD8:CD4 ratio. • PitNETs with higher Ki-67 expression had more FOXP3^+^ T cells.	Marques et al., 2019a
• The distributions of immune cells differed between PitNETs and normal pituitary tissues and varied among PitNET subtypes. T cells dominated the immune microenvironment across all subtypes of PitNETs.	Wang et al., 2020
• Cushing pituitary tumors, both overt and subclinical cases, had higher CD8^+^ T-cell fractions than GH tumors, prolactinomas, hyperthyroid tumors, and silent tumors.	Yeung et al., 2020
**Other immune cells**	
• Except for ACTH adenomas, B cells and CD4^+^ T cells and CD8^+^ T cells were clustered in one group in PitNETs. • Immune cells (B cells, CD8^+^ T cells) showed a higher abundance in GH adenomas than in NF-PitNETs.	Zhou et al., 2020
• Deconvolution analysis identified potential mononuclear cell infiltrate in PitNETs, consisting of dendritic cells (DCs),* NK and mast cells.	Taniguchi-Ponciano et al., 2020
• NF-PitNETs secreted more cytokines and had 9 times more neutrophils than somatotropinomas.	Marques et al., 2019a

*Dendritic cells are the most functional antigen-presenting cells, named after their many dendritic or pseudopodial-like protrusions that protrude during maturation.

Lu et al. (2015) reported that CD68^+^ macrophage infiltration was present in all PitNETs. Additionally, the number of CD68^+^ macrophages was found to be higher in sparsely granulated growth hormone (SG-GH) adenomas and null cell adenomas than in densely granulated growth hormone (DG-GH) adenomas or ACTH adenomas. Principe et al. (2020) reported that gonadotropin (Gn) adenomas showed a higher degree of CD68^+^ macrophage infiltration compared to GH adenomas, PRL adenomas, and ACTH adenomas. Existing reports consistently suggest that the predominant subtype of macrophages in PitNETs is the M2 subtype. Interestingly, studies have shown that NF-PitNETs have a higher infiltration of M2 macrophages than other types of PitNETs.

Current researches suggest that the majority of PitNETs exhibit lymphocyte infiltration, including CD45^+^ T cells, CD3^+^ T cells, CD4^+^ T cells, and CD8^+^ T cells (Lu et al., 2015; Marques et al., 2019a; Principe et al., 2020; Wang et al., 2020; Zhou et al., 2020). Recent studies based on RNA deconvolution have reported a higher proportion of CD8^+^ T-cell infiltration in ACTH adenomas than in GH adenomas, PRL adenomas, TSH adenomas, and nonfunctioning NF-PitNETs (Yeung et al., 2020). In another recent study, a distinct pattern of immune infiltration was observed PitNETs. It was found that PitNETs with abundant macrophage and NK cell infiltration tended to have limited infiltration of B cells, CD4^+^ T cells, and CD8^+^ T cells, and vice versa (Zhou et al., 2020).

In general, functional PitNETs exhibit a higher percentage of immune cell infiltration than NF-PitNETs. Immune cell infiltration varies among different functional PitNETs. GH adenomas show higher infiltration of B cells and CD8^+^ T cells, Gn adenomas exhibit increased infiltration of CD68^+^ macrophages, while ACTH adenomas display elevated infiltration of CD8^+^ T cells. Within the same type of PitNETs, different pathological subtypes can result in varying levels of immune cell infiltration. For example, SG-GH adenomas have higher infiltration of CD68^+^ macrophages than DG-GH adenomas. Therefore, immune cells within the TME of functional PitNETs are likely to play a more prominent role in regulating biological behavior, growth, and invasiveness than those in the TME of NF-PitNETs.

### 4.3 The cytokine profiles in the TME of PitNETs

Tumor microenvironment contains abundant cytokines that may influence the growth and survival of tumor cells, regulate ECM remodeling, modulate tissue inflammation responses, and stimulate neovascularization. Most studies supported the role of cytokines in promoting hormone secretion, cell proliferation, and invasiveness in PitNETs. Some of these cytokines include interleukin-6 (IL-6) (Feng et al., 2016; Wu et al., 2016), interleukin-1 (IL-1) (Gong et al., 2005; Haedo et al., 2009; Vindeløv et al., 2011), interleukin-2 (IL-2) (Arzt et al., 1995), interleukin-17 (IL-17) (Qiu et al., 2011, 2013), interleukin-22 (IL-22), and tumor necrosis factor-alpha (TNF-α) (Wu et al., 2016; Zhu et al., 2018).

Interleukin-6 is an important cytokine involved in the development and invasiveness of pituitary tumors. Multiple pieces of evidence suggest that IL-6 and its receptors are highly expressed in PitNET cells and tumor-associated fibroblasts (TAFs) (Kurotani et al., 2001; Haedo et al., 2009; Marques et al., 2019a,b). IL-6 may play a role in determining the degree of tumor invasiveness (Feng et al., 2016; Wu et al., 2016; Marques et al., 2019b). Additionally, IL-6 may contribute to the secretion of GH in GH-secreting adenomas, or be involved in the high ACTH secretion and chronic inflammatory state in ACTH-secreting adenomas (Paoletta et al., 2011; Shah et al., 2017). Ueland et al. (2009) found significantly elevated levels of IL-1β in the serum of patients with active acromegaly caused by GH-secreting adenomas. Studies by Kovalovsky et al. (2004), Gong et al. (2005), and Vindeløv et al. (2011) demonstrated that IL-1 stimulates ACTH and GH secretion in animal pituitary tumor cells (AtT-20 and GH3 cells), as well as in human GH-secreting adenomas. Cannavo et al. (2014) reported high expression of IL-22 in the serum of PitNET patients, with PRL-secreting adenoma patients showing higher levels of IL-22 than NF-PitNET patients. In a study investigating the serum levels of IL-17 in PitNET patients before and after surgery, it was found that patients with invasive PitNETs had higher levels of IL-17 before surgery, and decreased levels after surgery. Furthermore, patients who had complete tumor resection had lower levels of IL-17 than patients with residual tumors after surgery (Qiu et al., 2011, 2013). These data suggest that the aforementioned cytokines may contribute to the occurrence and development of PitNET invasion.

Tumor necrosis factor-alpha is associated with the invasion and progression of PitNETs. Wu et al. (2016) conducted a comparative immunohistochemical study and found that invasive PitNET samples showed higher expression of TNF-α than noninvasive PitNET samples. Another study confirmed elevated TNF-α expression in PitNETs with bone invasion. *In vitro* experiments have shown that TNF-α induces osteoclastogenesis in PitNETs, leading to bone destruction (Zhu et al., 2018). Xiao et al. (2011) reported that the stimulation of mice with TNF-α resulted in hemorrhagic changes in xenografted PitNETs and enhanced the expression of vascular endothelial growth factor (VEGF) and matrix metalloproteinase-9 (MMP-9) in tumor vasculature. Therefore, it is supposed that TNF-α may play an important role in tumor bleeding in PitNETs by upregulating VEGF and MMP-9.

Leukemia inhibitory factor (LIF) expression can be detected in both normal pituitary tissue and PitNETs (Haedo et al., 2009). This cytokine regulates cell proliferation, differentiation, and phenotype. Specific LIF binding sites have been identified in human ACTH cells and mouse pituitary tumor AtT-20 cells (Stefana et al., 1996). LIF inhibits the growth of tumor cells by blocking the cell cycle (Stefana et al., 1996). In terms of hormone secretion, LIF stimulates ACTH cell secretion (Stefana et al., 1996) and inhibits the secretion of PRL and GH in rat pituitary MtT/SM cells (Tomida et al., 2001). In a study involving 98 PitNETs, it was observed that approximately 92% of samples expressed LIF. Notably, NF-PitNETs exhibited substantially higher LIF immunohistochemical scores than functional PitNETs (Kontogeorgos et al., 2000). In the TME, the cytokine interferon-γ (IFN-γ) is associated with hormone secretion inhibition and cell proliferation suppression in PitNETs (Haedo et al., 2009). It has been demonstrated that IFN-γ inhibits tumor proliferation and hormone secretion by suppressing the JAK-STAT1/NF-κB pathway in human ACTH tumor cells and mouse AtT-20 cells (Labeur et al., 2008).

### 4.4 The growth factor profiles in the TME of PitNETs

Growth factors (GFs) are a class of polypeptide substances that exert various biological functions by binding to specific cell surface receptors. These functions include regulating cell differentiation, altering the proliferation rate, and affecting structural functionality. In pituitary cells, multiple GFs are secreted, including transforming growth factor-beta (TGF-β), VEGF, fibroblast growth factor (FGF), and macrophage colony-stimulating factor (M-CSF). These GFs play a critical role in regulating the growth of pituitary cells. By interacting with cell surface receptors, they may modulate cell proliferation, differentiation, and function, thereby maintaining the normal physiological functioning of pituitary cells.

Due to the higher upregulation of TGF-β1 than of TGF-β2 or TGF-β3 in tumors, TGF-β1 has been a focal point of research in the field of oncology (Bierie and Moses, 2006). Interestingly, in a study investigating the TGF-β signaling pathway, the expression of TGF-β1 mRNA was found to gradually decrease from normal anterior pituitary lobe tissue to noninvasive NF-PitNET tissue, and further down to invasive NF-PitNETs tissue (Zhenye et al., 2014). Kim and Kim (2019) also reported a downregulation of the TGF-β signaling-related genes TGF-βR2 and TGF-β in invasive NF-PitNETs, suggesting that TGF-β1 may serve as an inhibitory factor for the growth and invasion of NF-PitNETs. Besides that, previous studies have indicated that platelet-derived growth factor-1 (PDGF-1) mimetics, such as ABT-510 and ABT-898, may inhibit the growth of PRL adenomas by increasing the activation of TGF-β1 in the pituitary. This suggests that the activation of the TGF-β signaling pathway may have an inhibitory effect on the occurrence of invasion in NF-PitNETs.

Vascular endothelial growth factor is expressed at higher levels in PitNETs than in normal pituitary tissue (Komorowski et al., 2000; Lohrer et al., 2001; McCabe et al., 2002; Borg et al., 2005; Marques et al., 2019a). This is particularly evident in dopamine receptor agonist-resistant PRL adenomas or in invasive PitNETs, where VEGF expression levels are significantly elevated (Sánchez-Ortiga et al., 2013; Sato et al., 2019). High expression of VEGF increases the risk of hemorrhage and stroke within PitNETs and contributes to the development of cystic changes in the tumor (Fukui et al., 2003; Gupta and Dutta, 2018). Furthermore, the balance between VEGF and other proangiogenic factors, such as FGF-2 and LIF, plays a crucial role in determining the differences in angiogenesis between normal pituitary tissue and PitNETs (Ferrara et al., 1987, 1992; Ferrara and Henzel, 1989; Leung et al., 1989; Turner et al., 2003). VEGF, in particular, induces the expression of the antiapoptotic protein Bcl-2, promotes endothelial cell proliferation, and prolongs endothelial cell lifespan, thereby facilitating angiogenesis in PitNETs (Nör et al., 1999). When conducting immunohistochemical analysis of M-CSF in PitNET samples, it was observed that recurrent PitNETs exhibited significantly increased expression of M-CSF compared to primary PitNETs. In addition, high concentrations of M-CSF receptor inhibitors have been shown to inhibit cell proliferation in mouse PitNETs (Matsuzaki et al., 2023). TAFs in PitNETs are important sources of FGF. Research conducted by Marques et al. (2019b) has demonstrated that FGF can stimulate the growth and secretion of various cytokines, such as IL-6, by TAFs. This process leads to increased invasiveness of PitNETs, alterations in vascular formation, and the induction of epithelial-mesenchymal transition (EMT). Therefore, in addition to TGF-β, most growth factors in the TME in PitNETs may promote tumor proliferation, angiogenesis, and invasion.

### 4.5 The chemokine profiles in the TME of PitNETs

Chemokines are a class of small molecular proteins that induce directed migration of responsive cells by interacting with cell surface receptors. They can interact with receptors in the TME, recruit different subpopulations of immune cells, and influence the invasion and metastasis of tumor cells. Based on the positioning of the first two cysteine residues in the protein sequence, chemokines can be classified into four main categories: CC-chemokines, CXC-chemokines, C-chemokines, and CX3-chemokines (Griffith et al., 2014).

The chemokine CXC ligand 12 (CXCL12), also known as stromal cell-derived factor 1 (SDF-1), exerts a strong chemotactic effect on lymphocytes. Normal pituitary tissue does not express both CXCL12 and its receptor CXCR4 simultaneously; however, in PitNETs, both CXCL12 and CXCR4 are highly expressed (Barbieri et al., 2008; Grizzi et al., 2015). In a study using flow cytometry, it was observed that invasive PitNETs had a higher proportion of cells that were double-positive for CXCR4 and CXCL12 than noninvasive PitNETs (Xing et al., 2013). Another study has shown that the expression of CXCL12 was correlated with the vascular density of PitNETs. Under hypoxic conditions, CXCL12 mediated the mobilization of endothelial progenitor cells toward vascular endothelial cells, thereby promoting tumor angiogenesis (Nomura et al., 2009). Moreover, *in vitro* experiments conducted with rat and human pituitary tumor cells have provided evidence that CXCL12 stimulates tumor proliferation, DNA synthesis, and GH secretion (Florio et al., 2006; Massa et al., 2006; Lee et al., 2008). These findings suggest that the activation of the CXCL12/CXCR4 axis increases tumor angiogenesis, promotes tumor growth, and stimulates GH secretion in PitNETs.

In a study that examined the cytokine expression profile in tumor tissues of PitNET patients, it was found that all PitNETs express chemokine (C-X-C motif) ligand 8 (CXCL8) (Green et al., 1996). PitNETs with increased infiltration of macrophages and neutrophils were found to be associated with elevated levels of CXCL8 (Marques et al., 2019a). Previous studies have reported that CXCL8 plays a crucial role in immune cell chemotaxis within PitNETs (Waugh and Wilson, 2008; Coffelt et al., 2016; Zhang et al., 2016; Alfaro et al., 2017). RNA sequencing data from 7 recurrent and 23 nonrecurrent PitNETs identified significant differences in the expression of CXCL8 pathway genes (CXCL8, CXCR1, and CXCR2) (Salomon et al., 2018), indicating that CXCL8 may contribute to the invasive progression of PitNETs and resistance to conventional treatments.

In a recent study measuring the secretion levels of 42 different cytokines in the culture supernatant of PitNET primary cells from 24 patients, it was found that the highest secretion levels observed were chemokines, which are predominantly secreted by PitNETs, with a smaller proportion secreted by endothelial cells or stromal cells. The major chemokines identified were CXCL8, chemokine (C-X-C motif) ligand 2 (CCL2), chemokine (C-X-C motif) ligand 3 (CCL3), and chemokine (C-X-C motif) ligand 4 (CCL4) (Marques et al., 2019a). Accumulating researches have confirmed that PitNETs with higher infiltration of macrophages and neutrophils exhibit higher levels of CCL2, CCL3, and CCL4 secretion (Grattan-Smith et al., 1992; Mantovani et al., 2010; Alfaro et al., 2017). In addition, the degree of infiltration of CD8^+^ T lymphocytes is also correlated with increased levels of CCL2 and CCL4 (Marques et al., 2019a).

Compared to sporadic PitNETs, familial PitNETs exhibit higher expression of CCL5 in tumor tissues with abundant infiltration of macrophages. *In vitro* studies have shown that the upregulation of CCL5 leads to increased recruitment of macrophages and enhances the migration and invasive capabilities of tumor cells (Barry et al., 2019). Therefore, the interaction between PitNETs and CCL5 is considered to play a crucial role in the progression of PitNET invasion. Increased expression of CCL17 derived from macrophages is also associated with larger tumor volume and enhanced invasiveness of PitNETs. CCL17 promotes tumor invasion through the CCL17/CCR4/mTORC1 pathway (Zhang et al., 2021). In summary, the aforementioned studies indicate that PitNET cells can secrete various active chemokines, which promote the recruitment of macrophages, neutrophils, and lymphocytes, thereby regulating tumor behavior and enhancing PitNET invasiveness.

### 4.6 The interactions and biological functions among various components in the TME of PitNETs

In the TME, a wide range of immune cells and noncellular components surround and influence tumor cells. They may play different regulatory roles in the tumorigenic mechanisms of PitNETs, including tumor cell proliferation, migration, invasion, angiogenesis, ECM remodeling, and EMT activation.

The immune components and stromal components of the TME both play a role in the proliferation and invasion of PitNETs. In the normal pituitary gland, macrophages are primarily of the antitumor M1 phenotype. However, in PitNETs, protumor M2 macrophages are more predominant, as reported in previous studies (Marques et al., 2019a), and the significant increase in M2 macrophages is associated with PitNET invasion into the cavernous sinuses (Sato et al., 2019; Principe et al., 2020; Zhang et al., 2021). Furthermore, Yagnik et al. (2019) reported that in NF-PitNETs with cavernous sinus invasion, the M2:M1 macrophage gene expression ratio was greater than 1, whereas, in 80% of noninvasive NF-PitNETs, the M2:M1 ratio was less than 1. M2 macrophages in PitNETs are also associated with increased expression of matrix metalloproteinases (MMPs) and ECM remodeling (Principe et al., 2020; Zhang et al., 2021), indicating their potential role in enhancing tumor invasion and migration. In the TME, other immune cells also have effects on promoting or inhibiting PitNET proliferation or invasion. Sato et al. (2019) reported that invasive NF-PitNETs have a significantly higher FOXP3/CD8 ratio than noninvasive NF-PitNETs, suggesting that FOXP3^+^ T cells may play a role in promoting PitNET invasion. Iacovazzo et al. (2020) found that GH adenomas with cavernous sinus invasion have lower levels of CD8^+^ T cells than noninvasive GH adenomas, further supporting the antitumor role of CD8^+^ T cells.

Nonimmune cells also influence tumor proliferation, migration, and invasion. Invasive PitNETs exhibit higher expression of α-smooth muscle actin (α-SMA), VEGF, and CCL2 in TAFs, which can promote tumor cell proliferation, than noninvasive PitNETs (Lv et al., 2018). In another study, fibroblasts derived from PitNETs with cavernous sinus invasion were found to increase tumor cell migration and invasion through the secretion of IL-6 (Marques et al., 2019b).

Angiogenesis is the process of new blood vessel formation, and it plays a significant role in tumor growth and invasion (Vidal et al., 2001). However, most studies indicate that PitNETs have a lower microvessel density/area than the normal pituitary gland tissue (Jugenburg et al., 1995; Turner et al., 2000a; Vidal et al., 2000; Marques et al., 2020a). This may be a factor that contributes to the slow growth and low metastatic rate observed in PitNETs. Although the blood vessel density in PitNETs appears to be unrelated to their proliferation or invasion (Jugenburg et al., 1995; Turner et al., 2000b,^2003^; Vidal et al., 2000, 2001; Cristina et al., 2014; Marques et al., 2020a), there are differences in vascularization among different types of PitNETs. For example, NF-PitNETs have larger vessel calibers than GH adenomas (Marques et al., 2020a), which could be influenced by the TME. Previous studies have shown a positive correlation between the M2:M1 macrophage ratio and the microvessel density and microvessel area in PitNETs (Marques et al., 2019a,2020a). In a rat model of estrogen-induced PRL adenomas, the content of M2 macrophages increased as the capillaries became larger and more tortuous (Fujiwara et al., 2017). High expression of the angiogenesis marker VEGF-A was significantly correlated with the expression of CD163 (a marker for M2 macrophages), suggesting the potential involvement of M2 macrophages in angiogenesis. PitNETs with a higher presence of CD4^+^ T cells exhibited an increased microvessel area, while PitNETs with a higher presence of B cells displayed more rounded blood vessels. On the other hand, a higher presence of FOXP3^+^ T cells, CD4^+^ T cells, CD45^+^ T cells, and CD68^+^ macrophages was associated with lower microvessel density (Marques et al., 2020a; Mei et al., 2021), suggesting that these cells may also have an influence on angiogenesis. Additionally, the secretion levels of the cytokine CCL2 from PitNET cells were positively correlated with the blood vessel volume and microvessel area in PitNETs, indicating that CCL2 may play a positive regulatory role in angiogenesis in PitNETs (Marques et al., 2019b; Olson and Marks, 2019).

## 5 The relationships between the immune microenvironment and neuropsychiatric disorders and their underlying mechanisms

**FIGURE 1:**
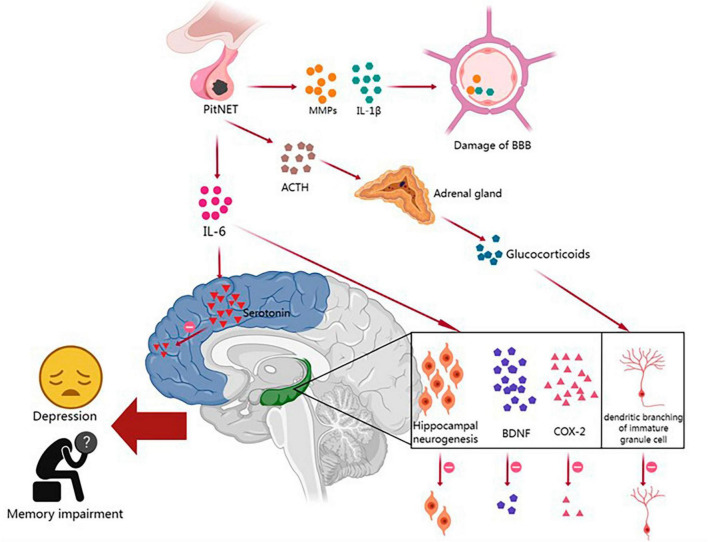
The relationships between the immune microenvironment and neuropsychiatric disorders in PitNETs and their potential mechanisms. PitNET, pituitary neuroendocrine tumor; MMPs, matrix metalloproteinases; IL-1β, interleukin-1β; IL-6, interleukin-6; BBB, blood–brain barrier; ACTH, adrenocorticotropic hormone; BDNF, brain-derived neurotrophic factor; COX-2, cyclooxygenase-2.

Pituitary neuroendocrine tumor patients have a higher prevalence of neuropsychiatric disorders such as depression, anxiety, and cognitive impairment than the general population, and these disorders significantly affect their quality of life and prognosis. Increasing evidence suggests bidirectional communication between the TME and the central nervous system (CNS) (Olson and Marks, 2019; Monje et al., 2020). Prolonged activation of the innate immune system can lead to the development of neuropsychiatric disorders. This process involves immune system activation-induced disruption of the blood–brain barrier (BBB) and chronic neuroinflammation, which can result in sleep disorders, abnormal glucocorticoid secretion, reduced neurogenesis in the hippocampus, decreased synthesis of monoamine neurotransmitters, and the dysregulation of neural network activity, thereby contributing to the development of depression and memory impairment (Figure 1).

The BBB is composed of endothelial cells, a basement membrane, and astrocytes, and it plays a critical role in protecting the brain parenchyma from infiltration by blood-borne cells and molecules (Kadry et al., 2020). Peripheral tumors can disrupt the BBB and affect brain function through the release of proinflammatory cytokines and the infiltration of immune cells (Olson and Marks, 2019). As mentioned earlier, PitNET patients exhibit increased expression of IL-1β and MMPs in the TME. Previous studies have shown that IL-1β can disrupt the integrity of the endothelial cell barrier by degrading and relocating tight junction proteins, while MMPs can further degrade the basement membrane and disrupt the tight junctions of endothelial cells (Wardill et al., 2016; Kadry et al., 2020). When BBB integrity is compromised in PitNET patients, solutes and immune cells from the periphery are more likely to enter the central nervous system, leading to the impairment of cognitive function.

Many cognitive and emotional impairments in non-central nervous system tumor patients may be attributed to chronic low-level systemic inflammation, which affects key brain structures involved in memory and emotion regulation, such as the hippocampus. Numerous studies have reported a significant correlation between increased tumor-related fatigue and depression with elevated expression of pro-inflammatory cytokines IL-1β and IL-6 mRNA in the cortex and hippocampus (Pyter et al., 2009; Yang et al., 2014; Norden et al., 2015; Santos et al., 2019). Tumors can induce neurofunctional impairments through various mechanisms, including IL-6-mediated inflammatory signaling, a reduction in hippocampal neurogenesis, and decreased levels of brain-derived neurotrophic factor (BDNF) and cyclooxygenase-2 (COX-2). The upregulation of IL-6 and IL-1β in PitNET patients suggests that the inflammation induced by PitNETs may play a role in hippocampal dysfunction, leading to neurofunctional impairments (Yang et al., 2014).

Hypothalamic-pituitary-adrenal (HPA) axis hyperactivity plays a key role in the pathogenesis of depression. Researchers have found a strong correlation between elevated plasma IL-6 levels and HPA axis dysfunction in tumor patients diagnosed with depression (Jehn et al., 2010). This disruption of the hypothalamic feedback system is believed to be due to the desensitization of glucocorticoid receptors (GRs) in the brain. IL-6 can promote GR desensitization by disrupting GR nuclear translocation and/or transcriptional function (Silverman and Sternberg, 2012). Prolonged activation of the HPA axis and chronic exposure to increased levels of glucocorticoids also have other pathological effects on the brain, such as reduced hippocampal volume and decreased dendritic branching of immature granule cells, leading to memory impairments (Dioli et al., 2019).

Inflammatory-induced alterations in metabolic pathways, such as the indoleamine 2,3-dioxygenase (IDO)-kynurenine and 5,6,7,8-tetrahydrobiopterin pathways, have been shown to lead to significant changes in the synthesis of neurotransmitters dopamine, serotonin, and norepinephrine and may be implicated in the development of depressive symptoms in cancer patients (Vancassel et al., 2018). For instance, in an animal experiment, tumor implantation induced depressive-like behavior in mice, along with increased levels of IL-6 and TNF-α in the brain, which corresponded to reduced dopamine activity in the striatum and decreased serotonin in the prefrontal cortex (Lebeña et al., 2014). These findings further support the notion that tumor-induced inflammation can lead to the dysfunction of the monoaminergic system, resulting in altered neural signaling in the affected brain regions. PitNET-related neuropsychiatric disorders can significantly impair patients’ quality of life, however, our current understanding of how the immune microenvironment of PitNETs influences critical brain functions remains limited. Therefore, it is crucial to conduct further research to comprehend the impact of peripheral tumors on brain homeostasis, and elucidate the molecular mechanisms by which the immune microenvironment of PitNETs induces psychological and cognitive impairments, thereby improving the quality of life and treatment outcomes for patients with PitNET-associated neuropsychiatric disorders.

## 5 Discussion

Patients with PitNETs often experience neuropsychiatric disorders due to factors such as impaired hormonal imbalances, and inadequate management of medications, surgeries, and radiation therapies. Commonly observed disorders include depression, anxiety, and cognitive dysfunction, which significantly impact patients’ quality of life and prognosis. Personalized comprehensive treatment may result in the improvement or recovery of neurobehavioral symptoms for some PitNET patients; however, these symptoms may be irreversible for certain patients. Therefore, in clinical practice, it is essential to not only develop appropriate treatment plans specifically targeting PitNETs but also to conduct comprehensive assessments of patients’ neurobehavioral status and identify the underlying causes of neuropsychiatric disorders. This approach enables effective prevention, timely diagnosis, and early intervention to mitigate the serious impact of neuropsychiatric disorders associated with PitNETs.

In addition, PitNETs have a significant presence of immune cells within the TME, predominantly macrophages and T lymphocytes. Other immune cells, such as B lymphocytes, neutrophils, or NK cells, are also present but in smaller quantities. These immune cells secrete a variety of cytokines, growth factors, and chemokines, which interact with each other to form both protumor and antitumor mechanisms. Together, they regulate the biological behaviors of PitNETs, including tumor initiation, proliferation, migration, invasion, and angiogenesis. Some aggressive PitNETs exhibit poor responses to surgical, radiation, and conventional drug treatments. Sustained research on the TME can provide a better understanding of the development and progression of PitNETs, thereby assisting in identifying biomarkers for diagnosing and predicting the invasive behavior of PitNETs, determining immunotherapeutic targets for refractory PitNETs, and appraising the specific populations that would benefit the most from immunotherapy.

This review provides a pioneering summary of the close relationships between the aberrant secretion of proinflammatory cytokines within the TME of PitNETs and the occurrence of neuropsychiatric disorders, along with their potential underlying mechanisms. The cytokines produced as a result of TME dysregulation may affect various aspects of the central nervous system, including neurotransmitter metabolism, neuroendocrine function, and neurovascular plasticity, thereby leading to a higher susceptibility to neuropsychiatric disorders in PitNET patients.

However, the current understanding of the mechanisms underlying the development of neuropsychiatric disorders in patients with PitNETs due to TME dysregulation is still limited. Further in-depth researches are required to explore the potential for alleviating neuropsychiatric disorders in PitNET patients through the modulation of TME dysregulation, thereby providing more substantive evidence. Currently, there is an urgent need to enrich diagnostic methods for neuropsychiatric disorders in PitNET patients, improve and update diagnostic criteria, as well as enhance the availability of intervention strategies to decrease the incidence of neuropsychiatric disorders associated with PitNETs, ultimately leading to an improvement in the quality of life for affected patients.

## Author contributions

SYC: Conceptualization, Investigation, Methodology, Visualization, Funding acquisition, Writing – original draft, Writing – review & editing. SNC: Conceptualization, Investigation, Methodology, Visualization, Writing – original draft, Writing – review & editing. XCW: Writing – review & editing. QW: Conceptualization, Funding acquisition, Investigation, Methodology, Project administration, Supervision, Visualization, Writing – original draft, Writing – review & editing.
